# Multiple indicators of rice remains and the process of rice domestication: A case study in the lower Yangtze River region, China

**DOI:** 10.1371/journal.pone.0208104

**Published:** 2018-12-03

**Authors:** Yongchao Ma, Xiaoyan Yang, Xiujia Huan, Yu Gao, Weiwei Wang, Zhao Li, Zhikun Ma, Linda Perry, Guoping Sun, Leping Jiang, Guiyun Jin, Houyuan Lu

**Affiliations:** 1 School of Archaeology and Museology, Peking University, Beijing, China; 2 Institute of Tibetan Plateau Research, Chinese Academy of Sciences, Beijing, China; 3 Key Laboratory of Cenozoic Geology and Environment, Institute of Geology and Geophysics, Chinese Academy of Sciences, Beijing, China; 4 Department of Archaeology and Natural History, Australian National University, Canberra, Australia; 5 School of Archaeology and Ancient History, University of Leicester, Leicester, United Kingdom; 6 School of Cultural Heritage, Northwest University, Xi’an, China; 7 The Foundation for Archaeobotanical Research in Microfossils, Fairfax, United States of America; 8 Department of Anthropology, George Washington University, Washington, DC, United States of America; 9 Zhejiang Provincial Institute of Cultural Relics and Archaeology, Hangzhou, China; 10 Institute for Cultural Heritage, Shandong University, Jinan, China; 11 University of Chinese Academy of Sciences, Beijing, China; 12 Center for Excellence in Tibetan Plateau Earth Science, Chinese Academy of Sciences, Beijing, China; University at Buffalo - The State University of New York, UNITED STATES

## Abstract

The process of rice domestication has been studied for decades based on changing morphological characteristics in assemblages of both macroremains, such as charred seeds and spikelet bases, and microremains, such as phytoliths, esp. bulliform and double-peaked phytoliths. The applicability of these indicators in determining if a specific assemblage is wild or domesticated, however, is rarely discussed. To understand the significance of these indicators in the determination of domestication, we collected 38 archaeological samples from eight Neolithic sites, dating from 10-2ka BP, in the lower Yangtze River region to analyze and compare the changes of these different indicators over eight thousand years. The data demonstrate that the comprehensive analysis of multiple indicators may be the best method to study the process of rice domestication developed thus far. An assemblage of rice remains can be identified as domesticated forms if they meet the following criteria simultaneously: 1) the proportion of domesticated-type bulliform phytoliths is more than 73%; and 2) the proportion of domesticated-type rice spikelet bases is higher than 75%. Furthermore, we found that each indicator tends to change steadily and gradually over time, and each stabilized at a different time, suggesting that the characteristics of domesticated rice developed slowly and successively. Changes of multiple indicators during the period between 10,000–2,000 yr BP indicate that the process of rice domestication in the lower Yangtze River region lasted as long as ca. 6,000 years during the Neolithic, and can be divided into three stages with the turning points in the middle Hemudu-late Majiabang culture (6,500–5,800yr BP) and the late Liangzhu culture (4,600–4,300yr BP).

## 1. Introduction

The origin and spread of rice agriculture has been studied and discussed since the 19^th^ century. Based on ancient documents and modern rice distribution, Emile V. Bretschneider from Germany, Alphonse Louis Pierre de Candolle from France-Switzerland, and Nicholas Ivanovitch Vavilov from the Soviet Union conducted initial studies on the origins of rice, and their findings have exerted significant influence on subsequent studies [1]. Rice is divided into two subspecies, *Oryza sativa* subsp. *japonica* and *Oryza sativa* subsp. *indica* [2]. In the 20^th^ century, rice remains recovered from Neolithic sites were all identified as *japonica* or analogous *japonica* in China, based on morphological features of charred seeds [3–13], bulliform phytoliths [14–20], and double-peaked phytoliths [8, 21–23]. Thus, the opinion that China is the domestication center of *japonica* is well supported and widely accepted [24–29]. This conclusion drawn from macro and micro rice remains is consistent with the results of genetic research as well [29–33]. Distinguishing between *japonica* and *indica*, therefore, is unnecessary when studying rice remains in Neolithic China.

**Fig 1:**
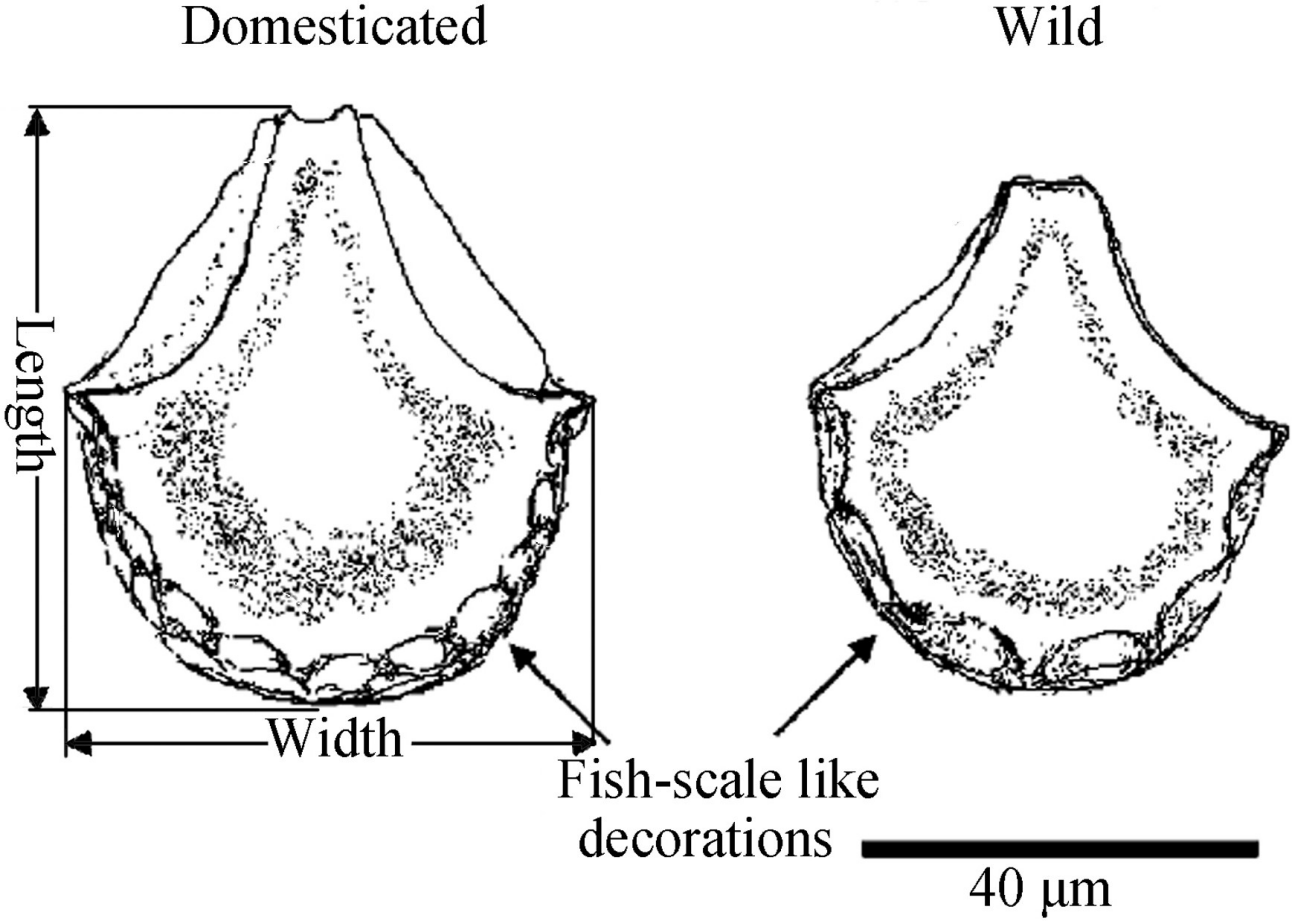
Parameters of rice bulliform phytoliths measured in the study (modified from [62]).

Distinguishing between wild and the domesticated rice remains from prehistoric sites and the processes of rice domestication have been studied and discussed since the transition to the 21^th^ century. The size of rice seeds, spikelet bases [34–38], fish scale-like decorations on bulliform phytoliths, and the size of double-peaked phytoliths [39] are used frequently, and each indicator reveals one facet of rice domestication. Due to the competition among individual seeds in the fields, the size of rice seeds tends to enlarge over time and in subsequent plantings [34,36]. Changes of the size of rice seeds and bulliform phytoliths also reveal the transition of rice seeds from immaturity to maturity, which is an important domestication marker [34, 36], though the latter could not be used to distinguish between wild and domesticated rice alone [40, 41]. As harvesting practices selected for certain traits, the tendency for seed shattering decreases, and is documented by the less and less wild type spikelet base with a smooth and round abscission scar and a small, distinct vascular pore [34, 36]. The fish scale-like decorations of the bulliform phytoliths (Fig 1) come into being due to the extrusion between bulliform cells and colorless cells in the epidermis of rice leaves, and the numbers of decorations are likely linked to the water supply and the paddy management. Wild rice grows in wetlands with perennial water inundation, and these plants have a lower probability leaf rolling which results in a smaller number of fish scale-like decorations. The irrigation and drainage of modern rice paddies increases the probability of leaf rolling and, thus, more fish scale-like decorations [42].

**Fig 2:**
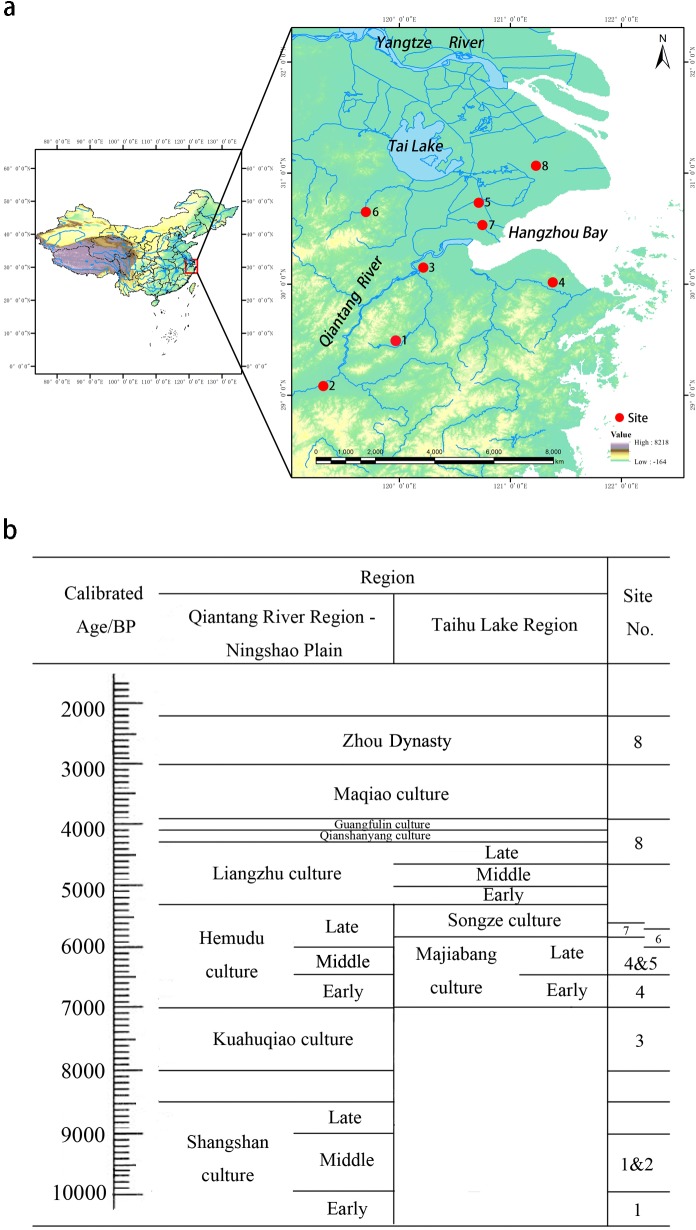
Locality of samples and the cultural sequence in the study region. 1. Shangshan site; 2. Hehuashan site; 3. Kuahuqiao site; 4. Tianluoshan site; 5. Majiabang site; 6. Anle site; 7. Xiantanmiao site; 8. Guangfulin site.

The lower Yangtze River region is considered to be one of the centers of rice domestication [32, 33, 43, 44] (Fig 2A). Because of the complete sequence for archaeological cultures here (Fig 2B) [39, 45–54] and well-preserved plant remains, the above-mentioned indicators can all be applied in the research of the processes of rice domestication in the lower Yangtze River region alone without taking other areas with controversial trajectories into consideration [34–39, 43]. For example, the analysis of rice remains from the archaeological sites of Shangshan, Kuahuqiao, and Tianluoshan shows that from the early Shangshan culture to the early Hemudu culture: 1) the length and width of rice seeds decreased first, then increased [55, 56]; 2) the proportion of bulliform phytoliths with ≥9 fish-scale decorations (Fig 1) increased gradually, then decreased [39, 43, 57]; and 3) the proportion of domesticated-type spikelet bases (hereafter, DTSB in brief) [37] and the proportion of domesticated-type double-peaked phytoliths (hereafter, DTDP in brief) increased gradually [39]. It is difficult to interpret the discrepancies derived from these different parameters.

Here we analyze the parameters of rice bulliform phytoliths, including length, width, and the proportion of bulliform phytoliths with ≥9 fish-scale decorations (refer to Fig 1) from a series of archaeological samples in the lower Yangtze River region (Fig 2), comparing the trajectories documented in these changing plant remains with the trends in relative proportions of DTSB, DTDP and seed size between 10-2ka BP. We evaluate the applicability of each indicator and assess the entire process of rice domestication for the region.

## 2. Material and methods

We collected 38 archaeological samples from 8 Neolithic sites in the lower Yangtze River region dating from ~10,000–2,000 BP (Fig 2, S1 Table), under permission of the superintendents of archaeological sites, Prof. Guoping Sun, Prof. Leping Jiang, Prof. Yunfei Zheng and Prof. Fen Wang. Samples were collected from cultural layers except the samples from Xiantanmiao site which were collected from pit fill.

The extraction of rice phytoliths was completed using the wet ashing method in the Key Lab. of Land Surface Pattern and Simulation, Institute of Geographical Sciences and Natural Resources Research, Chinese Academy of Sciences following Piperno and Lu [58, 59]. The experimental procedure is as follows: (1) 5 g samples are placed in a 50 ml test tube with 30% H_2_O_2_ for about 12 hours to destroy any organic material; (2) the samples are treated with 10% HCl and heated about half an hour to remove calcareous matter; (3) treated with 5% sodium hexametaphosphate ((NaPO_3_)_6_) for half an hour to disperse the clay; (4) floated with 2.35 g/cm^3^ heavy liquid (ZnBr_2_) to extract the phytoliths and rinsed by absolute ethyl alcohol and pure water successively; (5) the phytoliths are mounted with on a glass slide.

The samples are examined under a Zeiss optical microscope (magnification × 400) and rice bulliform phytoliths are identified from them according to Lu et al. [60]. The length, width and the number of fish-scale decorations of each are measured and recorded. The selection standard of bulliform phytoliths is based on Wang and Lu’s work [61]. They documented that the morphological differences of rice bulliform phytoliths from different positions in a piece of leaf or different leaves in a plant is very evident and the comparison of bulliform phytoliths from several leaves selected randomly does not reveal the complete picture. To ensure that the results were comparable, therefore, the asymmetrical phytoliths were excluded because they can be identified as being produced in a different part of the leaf, and only the symmetrical phytoliths were analyzed (S1A and S1B Fig). To ensure the accurate count of fish-scale decorations, poorly preserved phytoliths were excluded from this portion of the study (S1C–S1E Fig). Several layers of fish-scale decorations were observed on the edges of bulliform phytoliths in different microscope focal lengths, but only those on the outermost layer were counted. The proportion of the bulliform phytoliths with ≥9 fish-scale decorations, the domestication-type (hereafter, DTBP in brief) [62,63], was calculated. A minimum of 50 rice bulliform phytoliths were counted from each sample.

## 3. Results

Samples A-13 and A-14 from the Tianluoshan site represent the period of the middle Hemudu culture (6,500–6,000 BP), and samples A-15 and A-16 are from the layer of the late Majiabang culture (6,500–5,800 BP). The results from these four samples were analyzed together due to their same age, and the period is recorded as middle Hemudu-late Majiabang culture (hereafter, MH-LM culture in brief) in the following text. Samples A-1 and A-12 were characterized by poor preservation.

### 3.1 Length and width of rice bulliform phytoliths

**Fig 3:**
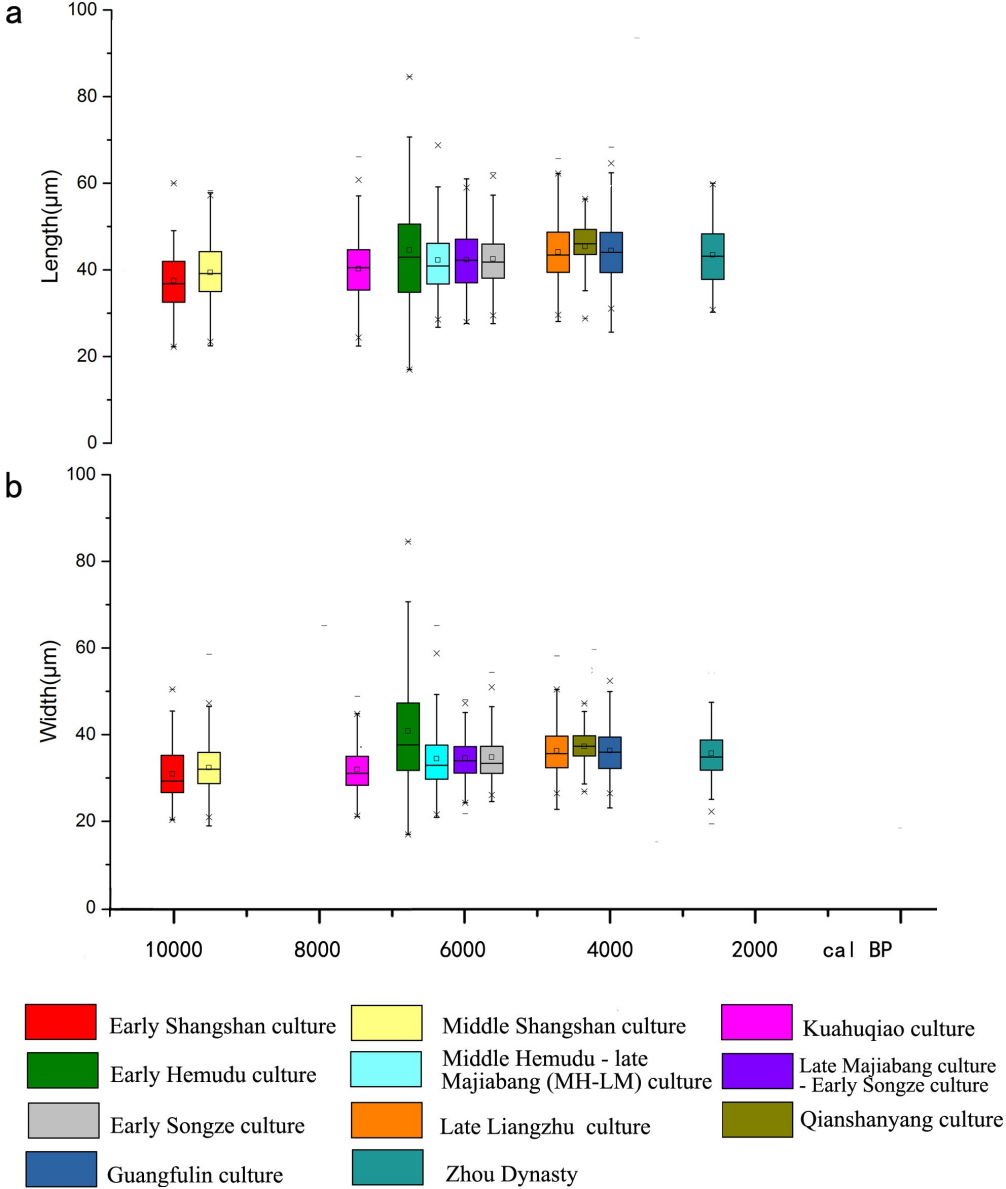
Changes of morphological features of rice bulliform phytoliths. a. Length; b. width.

A trend of increasing length and width of bulliform phytoliths recovered from archaeological samples was documented (Fig 3). The minimum mean of length and width are 37.48±6.92 μm and 30.90±6.21 μm, respectively, and occurred during the early Shangshan culture. From then on, both the length and width increased gradually and peaked during the early Hemudu culture with the mean values of 44.52±13.24 μm and 40.75±13.78 μm, respectively. During the MH-LM culture, both the length and width decreased while the mean length and width fluctuated around 42.00 μm and 34.50 μm until the early Songze culture. The length and width later increased slightly, and their mean value stabilized around 44.00 μm and 36.50 μm from the late Liangzhu culture to the Zhou Dynasty (Fig 3).

### 3.2 The proportion of DTBP

**Fig 4:**
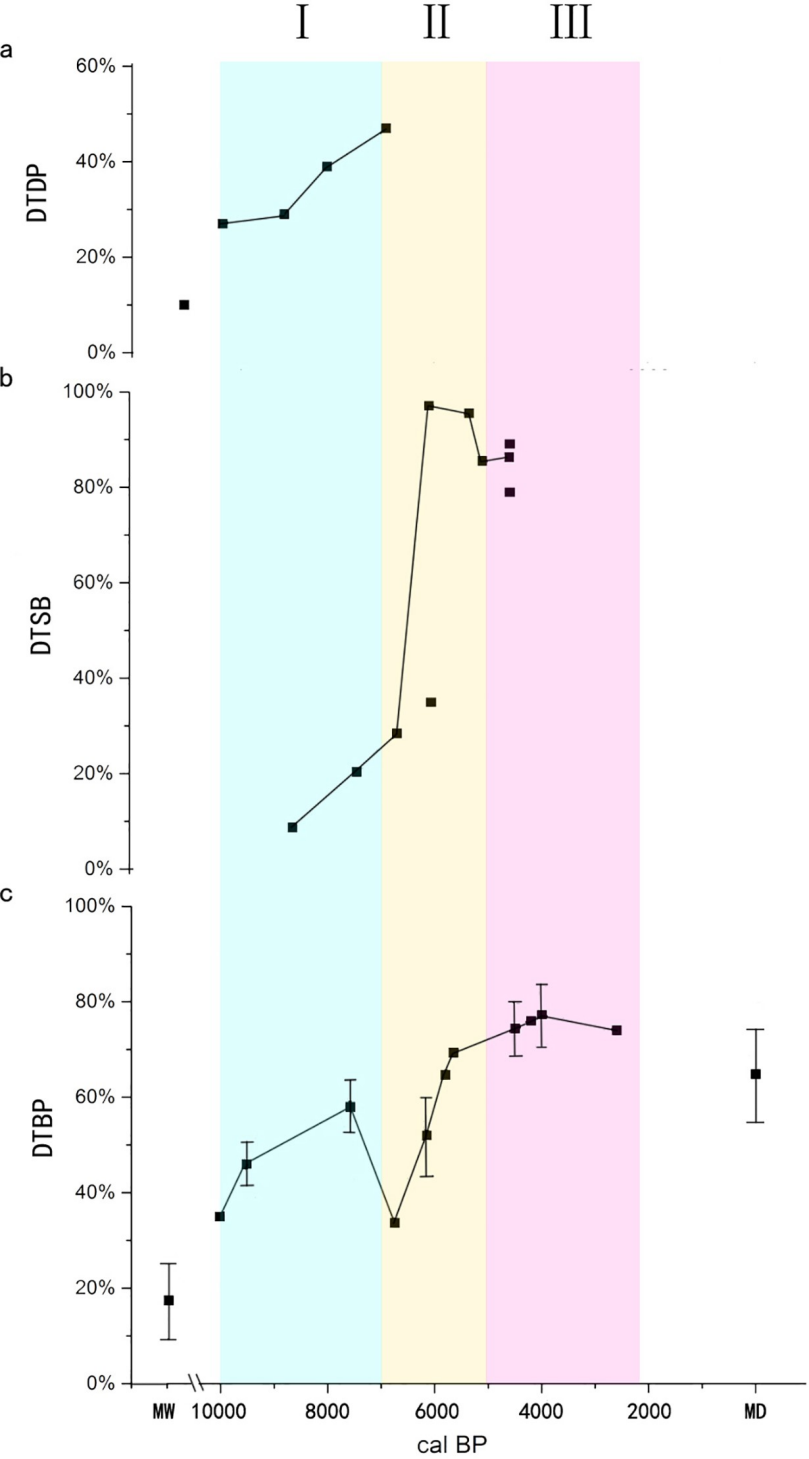
Trends of the proportion of DTDP, DTSB, DTBP. MW: modern wild samples; MD: modern domesticated samples. a. The trend of the proportion of DTDP; DTDP indicates rice domestication-type double-peaked phytoliths. The figure is modified from [39]. b. The trend of the proportion of DTSB; DTSB indicates domestication-type rice spikelet bases. c. The trend of the proportion of DTBP; DTBP indicates rice domestication-type bulliform phytoliths. The proportion of MW and MD is modified from [63].

The mean of the proportion of DTBP increased with time between the early Shangshan culture (34.98%) and the Kuahuqiao culture (58.00%±5.42%), and dropped to a minimum (33.82%) in the early Hemudu culture. The value rose from 52.00%±8.17% during the MH-LM culture to 69.33% during the early Songze culture, and stabilized during the late Liangzhu culture at a value of 74.40% ± 5.55%, as seen in Fig 4C.

## 4. Discussion

To uncover the entire process of rice domestication, other related data, such as the proportion of DTSB and DTDP and the size of rice seeds, are taken into account along with the phytolith data. Only previously published data collected and analyzed by professional archeologists is considered in the following discussion.

### 4.1 Comparison of parameters

#### 4.1.1 Rice bulliform phytoliths

Our data show that during the period of 10,000 to 6,000 BP, changes in the length and width were irregular, and from 5600 BP to 4600 BP, both parameters increased becoming stable after 4600 BP. This trend is similar to that documented in previous studies [24, 34, 42].

The comparison of the proportion of DTBP between the modern and the archaeological samples indicates that the proportion of DTBP is an applicable parameter for distinguishing wild and domesticated rice which could be used to depict trajectories in rice domestication. It has been demonstrated that the overall proportion of the wild type phytoliths to domesticated types differs between wild and domesticated populations of rice [63]. In our data set, the proportion of domesticated types during the early Shangshan culture (34.98%) is larger than in modern wild samples (17.46%±8.29%) [63]. Proportions of domesticated types in late Liangzhu culture (74.40%±5.55%), the Qianshanyang (76.00%) and Guangfulin cultures (77.25%±6.50%) are equal to or even exceed those of modern domesticated rice, demonstrating that rice had reached a modern domesticated level during the late Liangzhu culture (4,600–4,300 BP). Because the difference of the proportions of DTBP to wild types is related to the water supply [42], we conclude that the transition from flooded to drained fields [64] is probably responsible for the changes in phytolith proportions between the MH-LM culture and the early Songze culture.

#### 4.1.2 Rice spikelet bases

The published data available on spikelet bases were compiled and we calculated the proportion of DTSB using the method (the number of domesticated type/the total×100%) put forward by Fuller et al. [37] (Fig 4B). Zheng et al. [65] use a different method to study rice spikelet bases, dividing them into two types, a domesticated type and a wild type, with the former containing the immature type.

The calculated result from the Shangshan culture is 8.73% DTSB, based on the Huxi site [66]. The Kuahuqiao culture (8,000–7,000 BP) has a DTSB average of 20.85% according to an analysis from the Kuahuqiao site by Fuller et al. [38] and Zheng et al. [65]. The data concerning the proportion of DTSB for the early Hemudu culture is from Tianluoshan [67] with a calculated result of 28.43%. The values of MH-LM culture are 97.06% and 36.23% from the Majiabang [68] and Tianluoshan [67] sites, respectively, and the difference is much larger than that of the proportion of DTBP, which is 57.00% and 47.00%, respectively. The proportions of DTSB from the Songze and early Liangzhu cultures are 95.51% and 85.53% from the Xiaodouli [69] and Maoshan sites [68], respectively. The late Maoshan, Yujiashan and Liangzhu sites are all middle-late Liangzhu culture, and the proportion of DTSB are 86.32%, 89.46% and 75.41%, respectively [68]. The proportion of domesticated types in the Guangfulin culture is approximately 100% [68], but more data need to be collected to verify the value because of the limited material (Fig 4B). Changes of the proportion of DTSB suggest that the trend increased slightly between the Shangshan culture and the early Hemudu culture, then increased to the highest level and stabilized in the late Majiabang culture at the latest. The swift growth of the proportion of DTSB was related to the popularization of small paddy fields after 6500 BP [30], which effectively segregated wild and domesticated rice taxa [34, 36].

#### 4.1.3 Rice double-peaked phytoliths

Rice double-peaked phytoliths from the Shangshan, Kuahuqiao and Tianluaoshan sites were measured by Wu et al. [39] using the discriminant put forward by Zhao [70]. In terms of radiocarbon 14 dates, the results show: 1) the domestication process was very slow in the Shangshan culture; 2) the rate accelerated and the proportion of DTDP rose by 10% in the 800 years between the Shangshan culture and the Kuahuqiao culture; and 3) the rate decelerated and the proportion of DTBP rose by 6% in approximately 1000 years from the Kuahuqiao culture to the early Hemudu culture.

#### 4.1.4 The size of rice seeds

**Fig 5:**
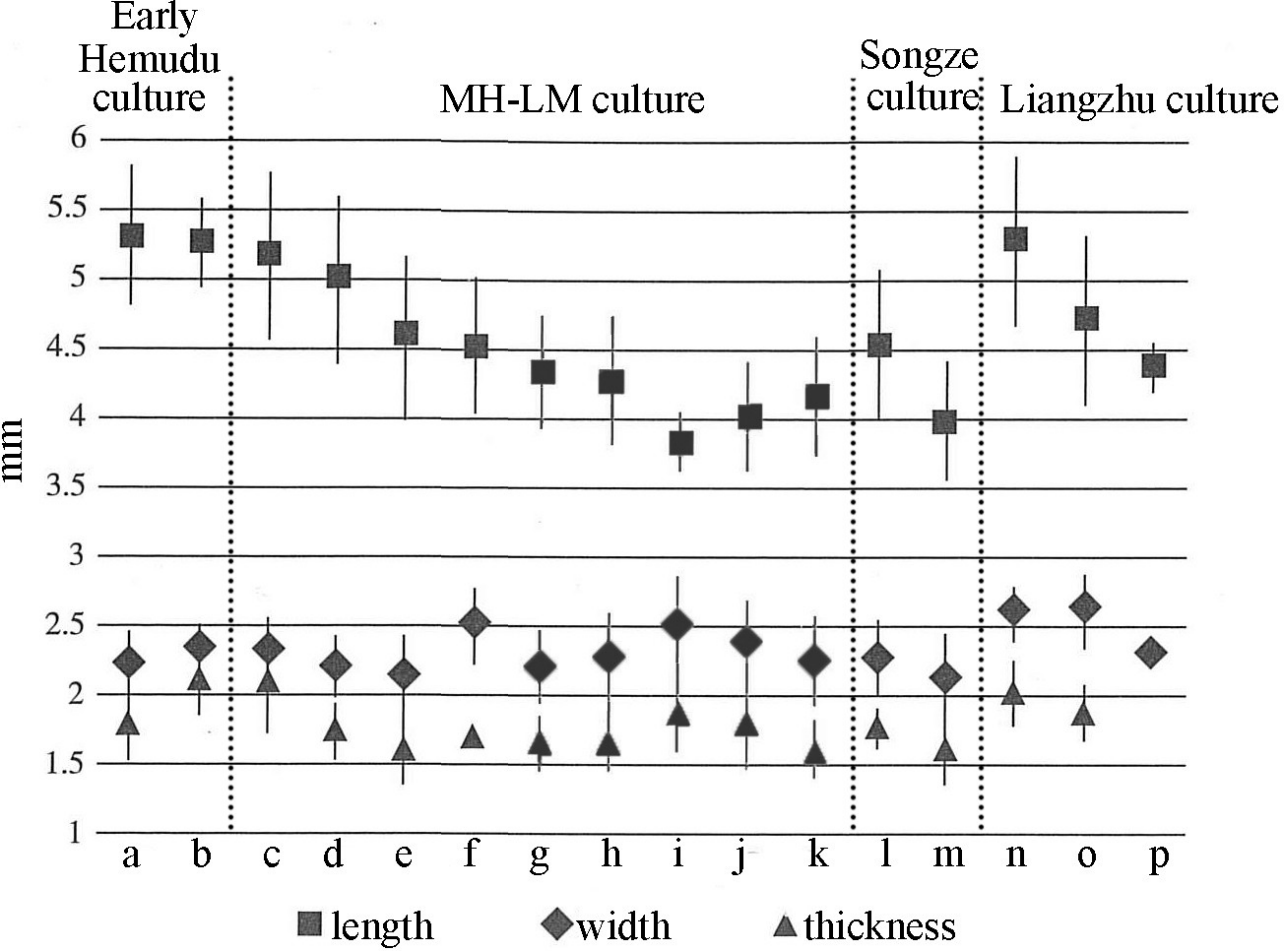
Changes of charred rice size over time (Modified from [55]). a. Tianluoshan site ⑧ (N = 80); b. Tianluoshan site ⑦ (N = 10); c. Tianluoshan site ⑥ (N = 88); d. Tianluoshan site ⑤ (N = 57); e. Tianluoshan site ④,③ (N = 124); f. Chuodun site (N = 96); g. Dongshancun site T2006 ⑭ (N = 105); h. Dongshancun site T2006 ⑬ (N = 99); i. Dongshancun site T1905 ⑫ (N = 9); j. Dongshancun site T1905 ⑪ (N = 107); k. Dongshancun site T2006 ⑩ (N = 15); l. Xiaodouli site (N = 6); m. Early phase of Maoshan site (N = 11); n. Yujiashan site (N = 10); o. Mojiaoshan site (N = 100); p. Jianshanwan site (N = 50).

According to the study carried out by Qin [55] on carbonized rice seeds from 8 Neolithic sites in the lower Yangtze River region, the period between the MH-LM culture and the Songze culture is the turning point in the trend of increasing size (Fig 5). For example, the length of rice from Dongshancun site decreased gradually between ⑭ and ⑫, then rose, similar to the trend documented at the Tianluoshan site. The trend of increasing size continued and stabilized at around 4.5 mm from the Songze culture to the Liangzhu culture. Changes of the width and thickness are not evident and measure around 2.5 mm and 1.75 mm, respectively. When these measurements are taken into consideration with changes of the size of bulliform phytoliths, we can conclude that the mature rice morphology was fixed around the time of the Songze culture (5800–5300 BP).

The comparison of the above parameters shows that the indicator employed to identify the rice remains is related to the age of site. During and before the MH-LM culture (6,500–5,800 BP), the changes in carbonized rice seeds, spikelet bases, and bulliform and double-peaked phytoliths are diverse. Thus, multiple indicators have to be used together during this period. No apparent changes of DTSB and the size of rice seeds occur in this period, so they are not applicable to use at sites of the Songze culture (5,800–5,300 BP). The focus of research should turn to the identification and distribution of the two species, *indica* and *japonica*, from the end of the Neolithic (4,300 BP at latest) when all the parameters stabilize and reach the modern domesticated level, including the size of bulliform phytoliths and the proportion of DTBP.

Previous studies [39, 40, 62] indicate that the proportion of DTBP is one of the indicators which stabilized most recently and is a credible index that can be applied alone to study the process of rice domestication. In addition, the proportion of DTBP in a phytolith assemblage is widely accepted as an indicator of domestication, and has been recently applied with relevant results [71, 72]. Based on this index, the first Neolithic domesticated rice remains in eastern Taiwan have been identified from the Chaolaiqiao site [73]; the apparent correlation between the exploitation of rice as a plant food resource and climate change has also been outlined in the northern Yangtze Delta [74].

### 4.2 The process of rice domestication in the lower Yangtze River region

Comprehensive analyses of the indicators discussed above suggest that rice domestication is not a singular event and the process is tripartite with turning points at the MH-LM culture and the late Liangzhu culture (Fig 3).

Stage Ⅰ of the rice domestication process occurs from the early Shangshan culture to the early Hemudu culture (around 10,000–6,500 BP). Changes in the indicators are inconsistent during this period and the rice domestication process advances slowly. The proportions of DTBP and DTDP in assemblages from the early Shangshan culture are higher than in modern wild levels (Fig 4). Thus, the beginning of the process of rice domestication can be identified as occurring around 10,000 BP or a little earlier [54]. From then on, the proportion of DTSB, DTDP and the length and width of bulliform phytoliths increase with time but at different rates. Changes in the size of rice seeds and the DTBP are not linear.

Stage Ⅱ of rice domestication occurs from the MH-LM culture to the early Songze culture (6,500–5,600 BP). Beginning with assemblages from the MH-LM culture, every indicator increased gradually at a rate that was higher than documented in the previous stage (Fig 4). The rice domestication process, therefore, accelerated with the popularity of small paddy fields, and we see the stabilization of the morphologies of the non-shattering spikelet, the size of the rice grain, and bulliform phytoliths. The proportion of DTSB increased by 19.7% in the 2000 years from the Shangshan culture to the early Hemudu culture, and then grew by 57.08% over the next 1500 years (from the early Hemudu culture to the Songze culture). The rate of the increase of the proportion of DTBP in the overall phytolith assemblage was 1.73%/100 years (17.33%/1000 years) between the MH-LM culture and the early Songze culture, which was higher than that from the early Shangshan culture to the Kuahuqiao culture, 0.37%/100 years (11.03%/3000 years). Moreover, the trend of increasing rice seed size changed during the MH-LM culture, especially the length, and the length and width of bulliform phytoliths decreased and stabilized.

Stage Ⅲ of domestication is from the late Liangzhu culture to the Zhou Dynasty (4,600–2,200 BP). Every indicator stabilizes, and the morphological characteristics remain stable into the Historic Age (Fig 4). The process of rice domestication can be concluded to have occurred over a period of at least 6,000 years.

## 5. Conclusion

This comprehensive analysis of the domesticated characteristics of rice seeds, rice spikelet bases, rice bulliform phytoliths, and rice double-peaked phytoliths demonstrates the following: 1) every indicator tended to stabilize at a particular time. The proportion of DTSB stabilized first during the MH-LM culture (6,500–5,800 BP); the size of rice seeds became stable during the Songze culture (5,800–5,300 BP); the length and width of bulliform phytoliths and the proportion of DTBP was fully shaped by the late Liangzhu culture (4,600–4,300 BP) at the latest. In other words, the domesticated rice characteristics emerged successively. 2) The process of rice domestication in the lower Yangtze River is lengthy, complicated, and lasts about 6,000 years. During the period of 10,000–6,500 BP, the process was slow, and even regressed. From 6,500 to 5,600 BP, the process accelerated, and from 4,600 to 2,200 BP, every indicator of domesticated rice remains was fixed at its modern level, and rice became fully domesticated.

At present, the systemic analysis of multiple indicators is the most feasible method to distinguish between wild and domesticated rice. If one assemblage of rice remains fits within the following criteria simultaneously, it can be confidently identified as domesticated: 1) the proportion of DTBP is more than 73%; and 2) the proportion of DTSB is larger than 75% provisionally. At this point we do not believe that the size of charred seeds and proportion of DTDP can be effectively used as criteria until more data are collected.

## Supporting information

S1 Fig Rice bulliform phytoliths recovered from archaeological samples. (TIF)

S1 Table Provenience, date, and rice data collected from the archaeological samples. (XLSX)
