# Supplementary figures and images for: Multiple indicators of rice remains and the process of rice domestication: A case study in the lower Yangtze River region, China
Source: PLoS One. 2018 Dec 3;13(12):e0208104. doi: 10.1371/journal.pone.0208104 (PMC6277086; doi:10.1371/journal.pone.0208104)

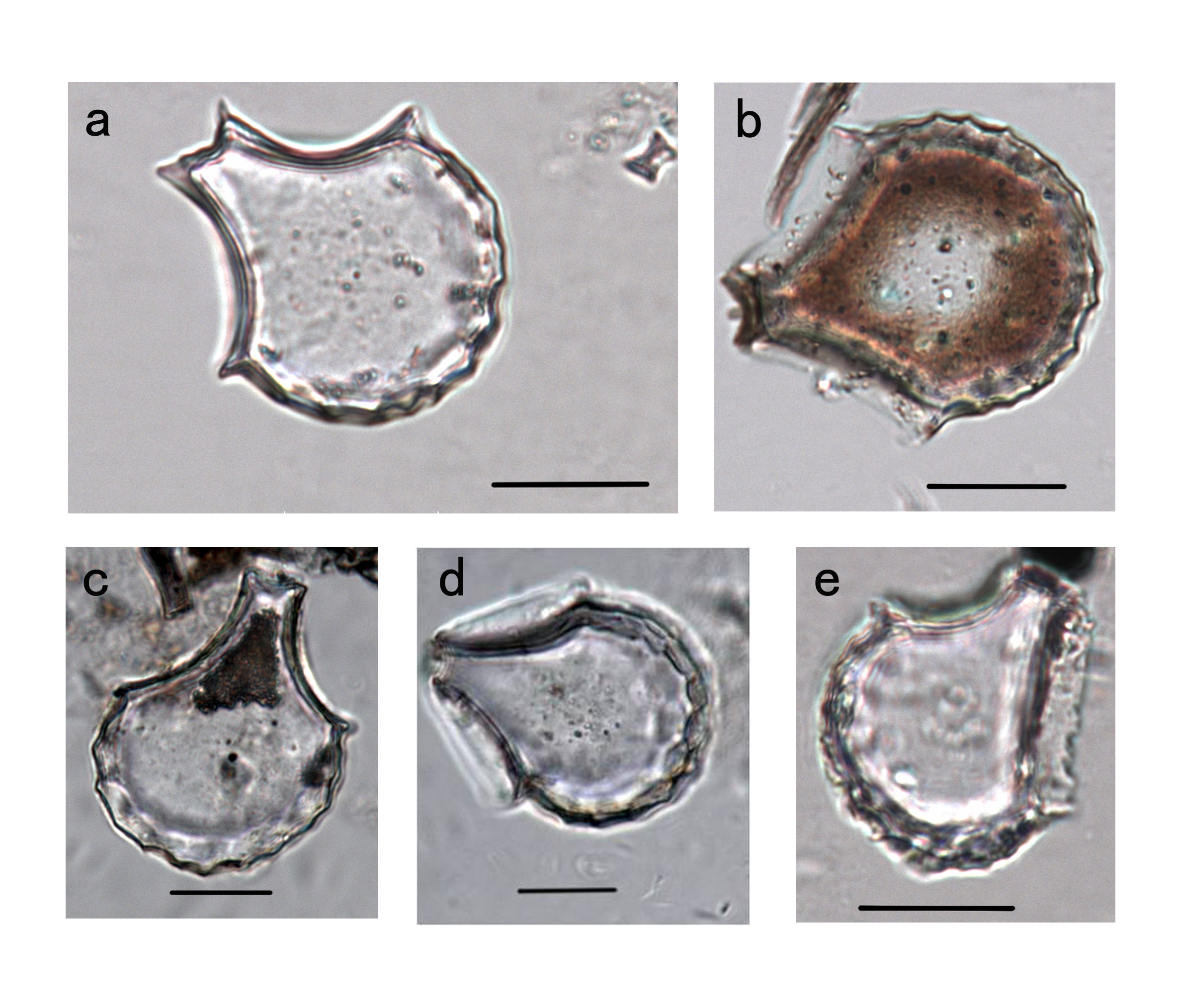

Supplement: S1 Fig — (TIF) [file pone.0208104.s001.tif]
